# A rabbit model for embolic infarct potentials of injectables using ultrasound-guided carotid artery puncture

**DOI:** 10.1038/s41598-022-21896-9

**Published:** 2022-11-10

**Authors:** Jiwoon Seo, Joon Woo Lee, Jungheum Cho, Eugene Lee, Heung Sik Kang

**Affiliations:** 1 College of Medicine, Seoul National University, Seoul, Korea; 2 Seoul Metropolitan Government-Seoul National University Boramae Medical Center, Seoul, Korea; 3 Seoul National University Bundang Hospital, 29 Gumi-ro 173 beon-gil, Bundang-gu, Seongnam-si, 13620 Gyeonggi-do Korea

**Keywords:** Biological techniques, Medical research, Risk factors

## Abstract

In order to evaluate the in vivo thrombogenicity of injectable agents, a suitable animal model is needed. We introduce an ultrasound-guided non-selective cerebral artery occlusion model via the common carotid arteries of rabbits. A total of 30 rabbits were assigned to an experimental group (n = 20) and a control group (n = 10). Each group received 2 mL suspension of embolic agent or 2 mL of normal saline, respectively, under ultrasound guidance. The animals were observed for immediate reaction and underwent magnetic resonance imaging (MRI) scan. Follow-up neurologic examination was conducted 24 h following the procedure. In 7 of the 30 rabbits, 2 in the control group and 5 in the experimental group, the administration of either normal saline or the embolic agent failed. Among the successfully injected 15 experimental animals, 14 showed neurologic impairment or deceased, whereas 1 animal did not show significant neurologic deficit. The MRI of 4 experimental animals showed detectable cerebral infarction on diffusion-weighted imaging. None of the 8 control animals showed neurologic abnormality and their brain MRI was normal. Our minimally invasive model is technically feasible and competent to show thrombogenecity of an injectable agent and consequent in vivo neurologic outcome.

## Introduction

Lumbar transforaminal and interlaminar epidural steroid injections are widely used to treat patients with radicular pain and radiculopathy. These therapeutic interventions for lumbar spine have been reported to be clinically effective for short- and long-term pain relief. Although this image-guided treatment is a minimally invasive technique, rare serious adverse events have occurred. Laemmel et al. have performed an in vivo study to elucidate the underlying mechanism of vascular obliteration induced by particulate steroids^1^. They found that several particulate steroids had an immediate and massive effect on microvascular perfusion, which was due to the formation of red blood cell aggregates associated with the alteration of these cells of into spiculated form. In 2011, the U.S. Food and Drug Administration announced a change in the safety labeling for triamcinolone acetonide, warning that triamcinolone acetonide “is not recommended” for epidural use and “spinal cord infarction, paraplegia, quadriplegia, cortical blindness, and stroke (including brainstem) have been reported after epidural administration^2^.”

In order to evaluate the safety and to assess the thrombogenicity of such agents, a preclinical animal model is necessary. Whilst several studies have attempted to generate suitable models, the conducted experiments demonstrated localized changes in vessels or tissues rather than neurologic or pathophysiologic outcomes^3–5^. Although, study of Laemmel et al. well demonstrated effect of a particulate steroid in in vivo environment but was designed to show change at femoral artery and did not reproduce neurologic outcome in the animal^1^. Silva et al. performed a study evaluating thrombogenic effect on venous structure of rat model. This study was also showed the localized effect on administered vein^4^. Furthermore, these studies required skin incisions and tissue dissections of experimental animals to approach the targeted vessels and to evaluate results. Thus, the development of a minimally invasive animal model that can reproduce neurologic outcomes is key for future investigations. However, to the best of our knowledge, no such animal model is available to date.

Therefore, the purpose of this study was to establish a preclinical model of non-selective cerebral artery embolization in rabbit to evaluate the thrombogenicity of injection agents with minimally invasive procedures.

## Methods

### Animals

Thirty female New Zealand white rabbits (weight range, 3.5−4.0 kg at the beginning of the study) were used. Rabbits were kept in individual cages in a humidity- and light-controlled (12-h light/12-h dark cycle) environment, temperatures were maintained at 21 ± 2 ℃, and animals were provided with standard laboratory chow and had ad libitum access to drinking water. All animal experiments were approved by the Regulations of Animal Care and Use Committees of Seoul National University Bundang Hospital (approval number: BA1608-206/050-01). All procedures were performed in accordance with the ARRIVE guidelines and the Guidelines for Animal Care and Use.

### Experimental design

Animals were divided into two groups based on randomization: (1) 20 rabbits were allocated to the experimental group, which received an injection of permanent embolic material, and (2) 10 rabbits were allocated to the control group and received the same volume of normal saline solution.

### Preparation of emboli

The embolic agent used consisted of 250−355-μm polyvinyl alcohol particles (Contour, Boston Scientific, Marlborough, MA, USA). The agent was suspended in a mixture of 10 mL of normal saline and 10 mL of nonionic contrast agent. A three-way stopcock was attached to the reservoir syringe to agitate the particles thoroughly prior to injection to avoid clumping of the particles.

### Neurologic examination

Each rabbit received neurologic examination before anesthesia and 24 h following the procedure, allowing adequate recovery from anesthesia. We adapted the four-point neurologic grading system used in the study of Endo et al., adding the maximum grade 5 corresponding to convulsive movement and death^6^. Neurologic deficits were scored by observing the animals on a smooth rubber mat on the flat floor. Symptoms such as paresis of the legs or abnormal gait (circling movement or difficulty walking) were observed: grade 1: no neurological deficit; grade 2: minimum or suspicious neurological deficit; grade 3: mild neurological deficit without abnormal movement; grade 4: severe neurological deficit with abnormal movement; grade 5: convulsive movement or death. This method has been successfully used previously in studies using rabbit models^7^.

### Anesthesia

All rabbits were premedicated with an intramuscular injection of 0.5 mg atropine and anesthetized with an intramuscular injection of alfaxalone (6 mg/kg) and xylazine (5 mg/kg) to the thigh. Anesthesia was maintained with a single intramuscular injection of alfaxalone (3 mg/kg) during transport from the laboratory to the Magnetic resonance (MR) scanner.

### Procedures

**Figure 1:**
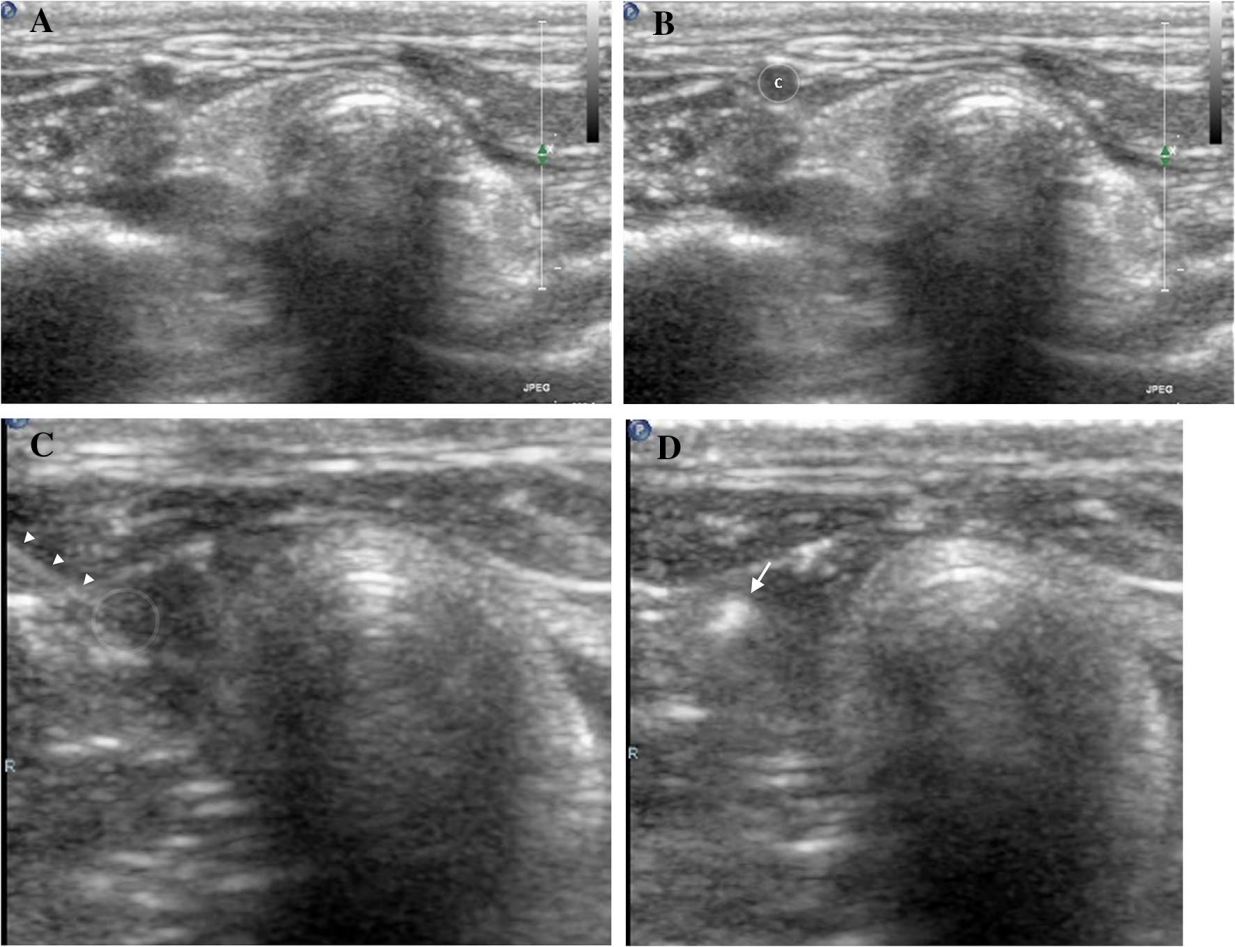
Puncture of the carotid artery and administration of the embolic agent. (**A**, **B**) The right common carotid artery (letter “C”) of the rabbit before puncture. (**C**) Puncture of the carotid artery with 23-gauge winged infusion set needle (arrowheads). (**D**) Monitoring of the embolic agent (arrow) administration.

Upon anesthesia, the fur of the anterior neck was removed using an electric clipper, from the sternal notch to the edge of the mandible (see Supplementary Fig. S1 online). The bilateral common carotid arteries were identified using the iU23 ultrasound scanner (Philips Healthcare, Bothell, WA, USA) and linear array transducer (L15-7io). The larger of the two carotid arteries was punctured using a 23-gauge winged infusion set under ultrasound guidance with a transverse approach. Once the needle was introduced into the arterial lumen, the embolic suspension or normal saline were administered after full regurgitation of blood to avoid an air embolism. Administration of the agent or saline was monitored with ultrasound (Fig. 1 and see Supplementary Video S2 online). In the experimental group, 2 mL of the embolic suspension were slowly injected over 5 s followed by 2 mL of normal saline. The control group was administered an injection of 4 mL of normal saline. After adequate compression of the carotid artery (for the prevention of hematoma and significant blood loss), the animals were observed for the immediate neurologic manifestation, such as convulsive movement or abnormal respiration, for 10 min.

In case of a puncture failure, the contralateral carotid artery was used for a second attempt after compression of the initially targeted artery. Puncture failure of both carotid arteries was considered as “puncture failure.”

### MR imaging acquisition

Rabbits were transferred to the MR unit using individual portable cages within 30 min after the injection. Using a 32-channel phased array sensitivity encoding head coil, MR imaging was performed on the anesthetized rabbits with a 3.0 Tesla Philips MR scanner (Ingenia, Philips Healthcare, Best, The Netherlands). Each rabbit was scanned in dorsal recumbency with the neck and head in an extended position. The rabbits were allowed to breathe spontaneously. The following parameters were used for acquisition of the multi-shot echo-planar imaging diffuse-weighted imaging (DWI), with image reconstruction using image-space sampling (IRIS) functions with b-values of 0 and 1000 s/mm^2^, and 3 orthogonal directions of diffusion gradients: echo time (TE) = 73 ms, repetition time (TR) = 6155 ms, slice thickness = 2 mm, interslice gap = 2.2 mm, number of averaging (NSA) = 2, bandwidth = 936 Hz/pixel, echo train length = 35, field of view (FOV) = 8.0 × 8.0 cm, and matrix = 80 × 80. In addition to DWI, oblique coronal turbo spin echo T2-weighted image (TR = 3000 ms, TE = 80 ms, NSA = 1, FOV = 8.0 × 8.0 cm, matrix = 80 × 80, slice thickness = 1.667 cm) and three-dimensional fluid attenuated inversion recovery images (TR = 4800 ms, TE = 438 ms, NSA = 2, FOV = 8.0 × 8.0 cm, matrix = 80 × 80, slice thickness = 0.8 cm) were obtained.

### Imaging interpretation

Two radiologists (with 8 and 14 years of experience, respectively) reviewed the MRI. Any of measurable conspicuous parenchymal lesions were assessed for the likelihood of infarction. A lesion with confirmative findings of infarction (hyperintense on DWI with corresponding low value on apparent diffuse coefficient map) was determined as true lesion, through consensus agreement.

### Statistical analyses

The normality of the distribution of the data was evaluated by using the Kolmogorov–Smirnov test. The Student’s *t-test* was used to compare the mean weight of the rabbits. *P* value < 0.05 was considered to indicate a statistically significant difference. Data were analyzed with SPSS software (version 22.0; IBM, Armonk, NY, USA).

## Results

### Ultrasound-guided carotid artery puncture

In 7 (23.3%) of the 30 rabbits included in this study, puncture of the bilateral common carotid artery failed. In control group, 2 (20%, 2 out of 10) cases were failed to be administered with normal saline. While, 5 (25%, 5 out of 20) cases from experimental group were failed to be administered with embolic agent. Necks of these seven rabbits with failed carotid artery puncture were properly compressed to prevent larger hematoma during procedure. All of them were well awake from anesthesia and showed no neurologic abnormality during 2 weeks of observation in cage.

The mean weight of rabbits in which the puncture failed was 3.41 ± 0.18 kg, whereas that of the successfully punctured counterparts was 3.75 ± 0.29 kg, which was significantly different (*P* = 0.019).

### Control group

All 8 control animals, successfully injected with normal saline, well awake from anesthesia. Their brain MRI did not show any signs of infarction. They did not show any neurologic deficit or sign of suffering during 2 weeks of observation in cage.

### Experimental group

**Figure 2:**
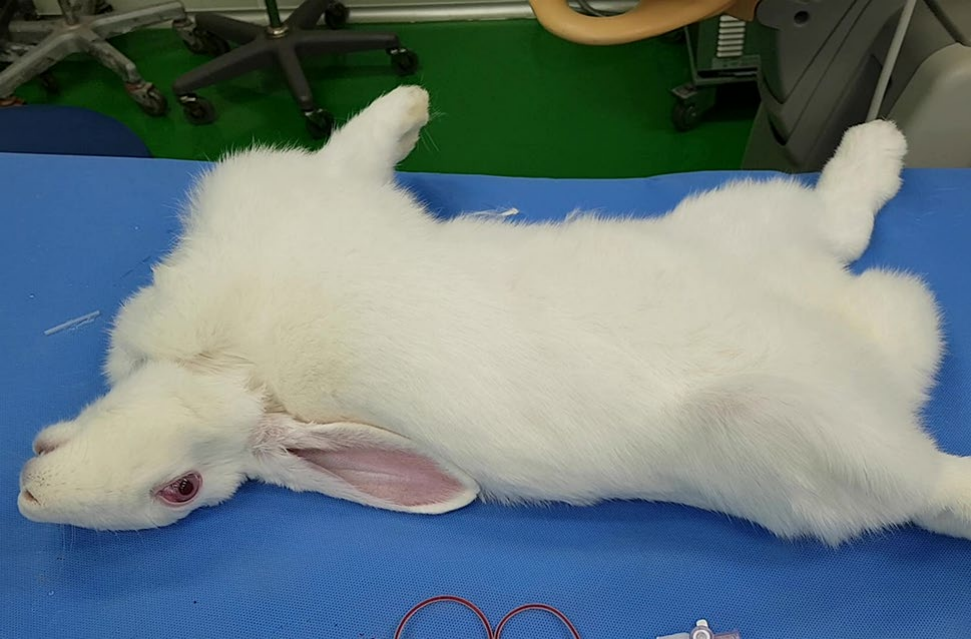
Rabbit showing convulsive seizure-like movement following administration of the embolic agent.

The neurologic outcome after 24 h following injection is summarized in Table 1. Among 15 successfully injected experimental animals, 14 rabbits showed grade 4 or greater neurologic deficits and 1 animal showed no deficits. Among the 12 rabbits with grade 5 neurologic deficits, 3 rabbits showed abnormal respiration, similar to Cheyne–Stokes breathing, and died immediately following the injection; another 3 died during the MR scan; the remaining 6 animals showed convulsive seizure-like movements (Fig. 2 and see Supplementary Video S3 online) soon after administration of the embolic agent. All animals with seizure-like movements died within 24 h following the injection. Two rabbits had hemiparesis showing abnormal gait (see Supplementary Video S4 online) and died 3 and 5 days after the experiment.

**Table 1:** Neurologic Outcome.

Grade	Experiment	Control
1	1	8
2	0	0
3	0	0
4	2	0
5	12	0
Failed puncture	5	2
Total	20	10

**Figure 3:**
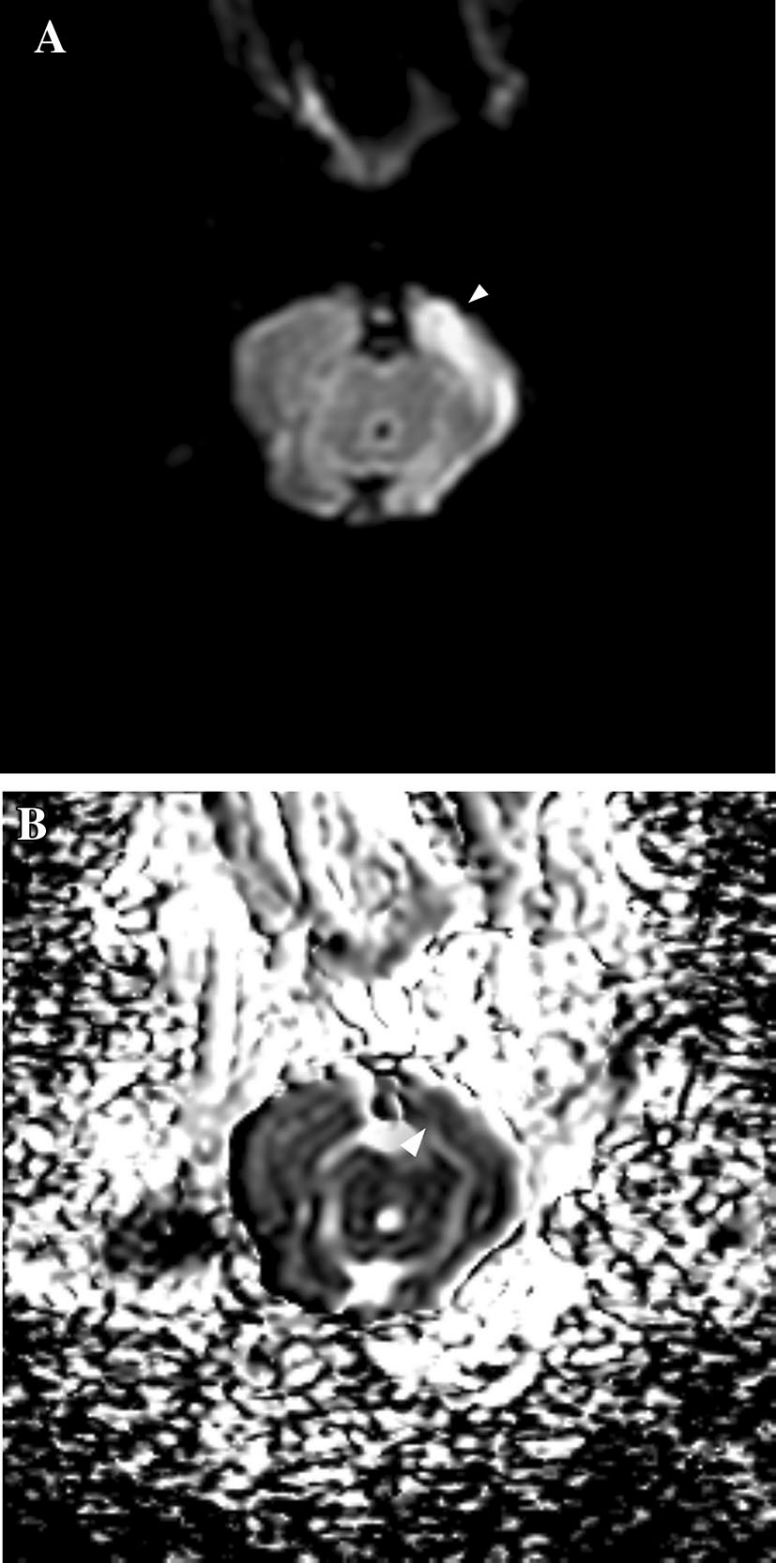
MR imaging of a rabbit showing an infarcted lesion. (**A**) Diffusion weighted imaging showing infarction (arrowhead) at the right temporal lobe. (**B**) Corresponding apparent diffusion coefficient map.

**Figure 4:**
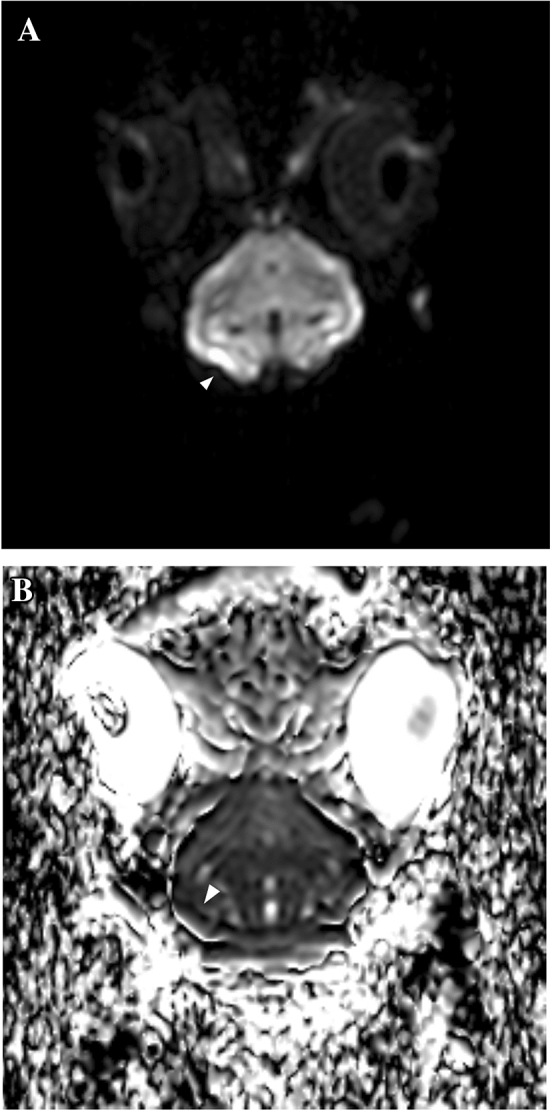
MR imaging of a rabbit showing an infarcted lesion. (**A**) Diffusion weighted imaging showing infarction (arrowhead) at the right parietal cortex. (**B**) Corresponding apparent diffusion coefficient map.

**Figure 5:**
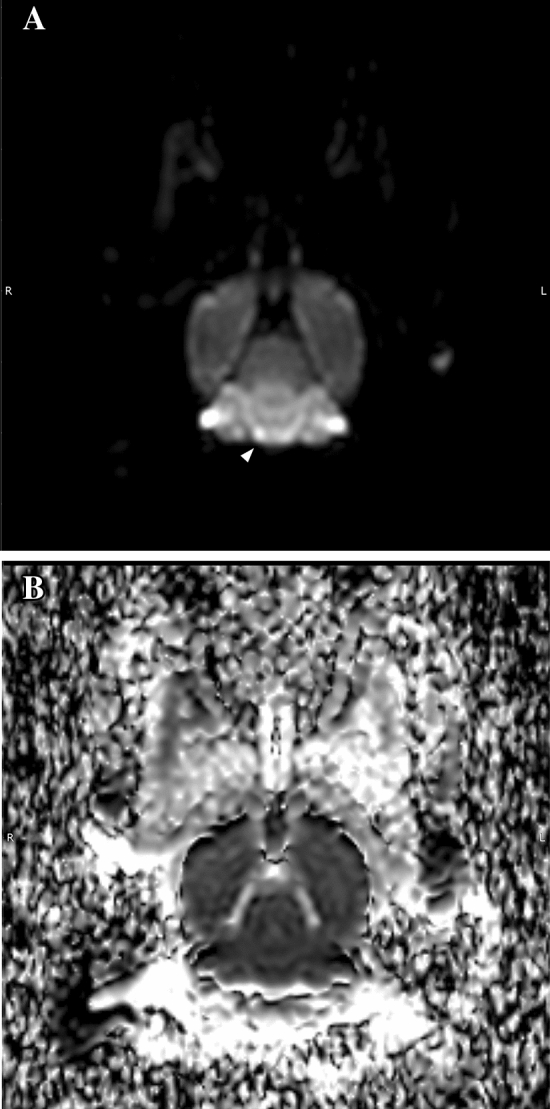
Magnetic resonance imaging of a rabbit showing a questionable infarcted lesion. (**A**) Diffusion weighted imaging showing suspicious infarction (arrowhead) at the right cerebellum. (**B**) Corresponding apparent diffusion coefficient map.

Excluding the rabbits that died before and during the MRI scan, 9 animals underwent a complete MRI scan. In 4 cases, the area of cerebral infarction was detectable and confirmative on DWI, with corresponding low value on apparent diffuse coefficient (ADC) map (Figs. 3 and 4). One rabbit had small hyperintense focus on DWI of the cerebellum, which was questionable for the true infarcted area (Fig. 5).

## Discussion

In this study, we developed and tested a new rabbit model to evaluate thrombogenicity of injectable agents under ultrasound guidance. As expected, the majority of rabbits showed severe neurologic deficits, whereas all control animals were unaffected.

Rare but serious adverse events, including brainstem, cerebellum, or spinal cord infarction, have been reported following epidural steroid injection, especially with particulate steroids^8–10^. There is also some experimental evidence corroborating these observed complications^1,11,12^. Radiculomedullary arteries are at risk of incidental puncture during epidural injection based on their anatomical proximity to the neural foramen, cervical through sacral Radiculomedullary arteries along with vertebral arteries and their anastomoses, can provide a direct anatomic pathway to the spinal cord, brainstem, or brain.

Some physicians have faith that particulate steroids are still more effective than non-particulate steroid, even though there is no clinical evidence proving their effectiveness. There have been several studies comparing these two types of steroids, although most of them failed to show statistically significant outcomes^13^. Nevertheless, patients treated with particulate steroids tend to have better self-reported outcomes and experience longer pain-free periods^14^. It was hypothesized that particulate steroid preparations may provide a local depot effect, with constant release of the active drug from the administration site over a longer time period compared to non-particulate steroids. An animal study performed by Abraham et al. used dogs to compare the duration of action of intramuscularly administered triamcinolone acetonide, a particulate glucocorticoid, to that of intravenously administered triamcinolone acetonide dihydrogen phosphate, a non-particulate steroid. They found that intramuscular administration of the particulate glucocorticoid had a duration of action of up to 4 weeks, which was much longer than a similar intravenous dose of its analogue, the non-particulate steroid^15^.

Unfortunately, following an announcement by the U.S Food and Drug Administration in 2011, triamcinolone acetonide, a particulate steroid, has been contraindicated for epidural injection for its serious and permanent complications, including blindness, stroke, paralysis, and death. Based on these circumstances, the development of novel forms of injection agents or drug vehicles was suggested, which can deliver steroids and have cumulative effects within the tissue for longer periods than non-particulate steroids, without any adverse effects. Whilst Laemmle et al. demonstrated the mechanism of arterial obliteration in their experiments, our study aimed to establish a preclinical model that can reproduce the intra-arterial reaction and evaluate the thrombogenicity of the injectable^3^.

There are several animal models that have been used to evaluate thrombogenic risks. Silva et al. used femoral veins of rats to elucidate the mechanism by which ultrafine particles interact with the vascular system^4^. Both femoral veins were exposed and cannulated, and the ear vein was observed for potential thrombosis following administration of rose bengal. This study used the venous system and required invasive procedures. A mouse carotid artery was used in a study by Kawasaki et al., demonstrating enhanced thrombogenicity of high Factor VIII plasma activity^5^. Although their study was aimed to assess intra-arterial reaction, a midline dissection needed for the exposure of the carotid artery of the mouse.

In contrast to these previous studies, our proposed thrombogenecity animal model does not require major invasive procedures, including skin incision, tissue dissection or wound closure. Any injection material can be administered by single puncture of carotid artery under ultrasound guidance and manifest its thrombogenic effect in cerebral artery. Since there is no surgical wound left on animals, these expected thrombogenic effect can be evaluated on immediate imaging or in-life neurologic examination. Immediate MRI scan following the injection would prevent or prevent sacrifice of the animal and allow long-term follow-up for the neurologic or functional outcomes.

**Figure 6:**
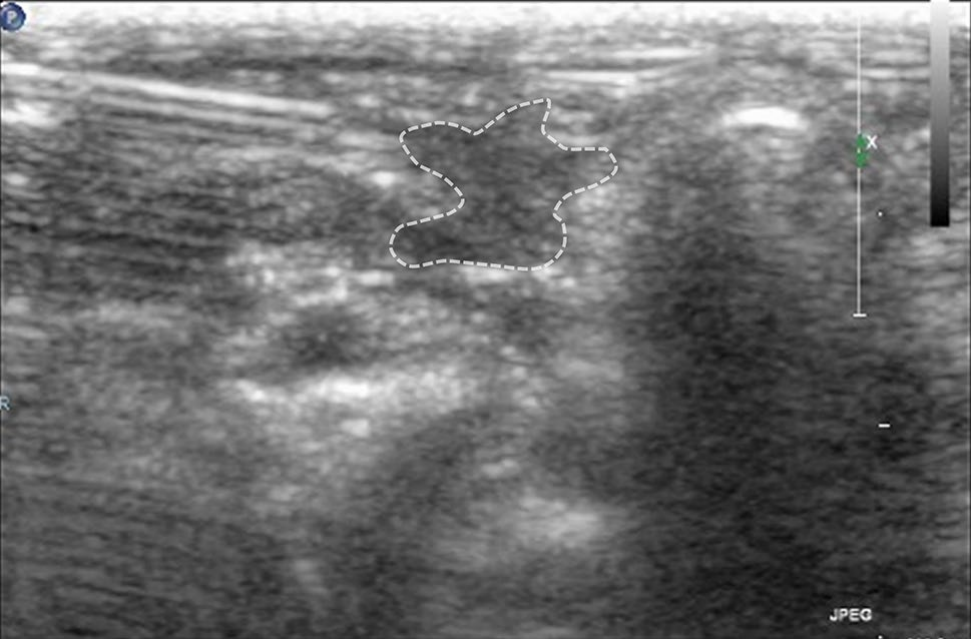
A large hematoma (dotted area) formation after failure of a puncture attempt.

There are several limitations to our study. First, there were puncture failures (23.3%). This was in part due to technical difficulties based on the relatively small size of rabbit carotid arteries compared to the human vascular structure. In fact, 5 cases of failure occurred in initial 15 rabbits and only 2 cases occurred among the subsequent 15 rabbits. It can be assumed that the puncture technique could be improved through repetition, considering the operator's learning curve. Moreover, in our retrospective analysis of animal factors, animals with puncture failure weighed significantly less than those in which the procedure was successful. We assume that the carotid arteries of lighter rabbits tend to be smaller in diameter and have a vulnerable arterial wall, which can be easily perforated with a slight touch of a needle tip (Fig. 6 and see Supplementary Video S5 online). Hence, we expect to lower failure rate by using bigger animal and technical improvement of an operator in future studies. Second, although we did observe neurologic deficits, not all rabbits showed significant positive findings on MR imaging. There are several possible explanations for these negative MR imaging findings. Most of the symptomatic rabbits underwent MR imaging within 2 h after administration of the embolic material. In the human brain, DWI is known to be less sensitive for detecting hyperacute infarction^16,17^. Moreover, some of the rabbits died before or during the MR scan, implying that other brain regions, apart from the infarcted area, could be affected by a lack in normal blood supply to rest of the brain parenchyma. Another potential explanation is the lack of an ideal MRI protocol for small animals. Although we used a protocol for rabbits devised by neuroradiology specialist and MR technician, sometimes diagnosis of infarction was challenged by unexpected artifact and suboptimal image quality. MRI protocol more suitable for small animals should be established in future studies. Further studies will be needed with different doses of embolic material that may be less fatal for the animals. Moreover, subsequent studies with particulate steroids are warranted.

In conclusion, we established a minimally invasive rabbit model for the evaluation of intra-arterial thrombogenicity under ultrasound guidance. This model is competent to show intra-arterial thrombogenetic effect of an injectable agent and consequent in vivo neurologic outcome. Further improvements in the imaging protocol will likely corroborate the usefulness of this new animal model.

## Supplementary Information

Supplementary Video 1.

Supplementary Video 2.

Supplementary Video 3.

Supplementary Video 4.

Supplementary Information 1.

Supplementary Information 2.
