# Supplementary figures and images for: A rabbit model for embolic infarct potentials of injectables using ultrasound-guided carotid artery puncture
Source: Sci Rep. 2022 Nov 10;12:19269. doi: 10.1038/s41598-022-21896-9 (PMC9649683; doi:10.1038/s41598-022-21896-9)

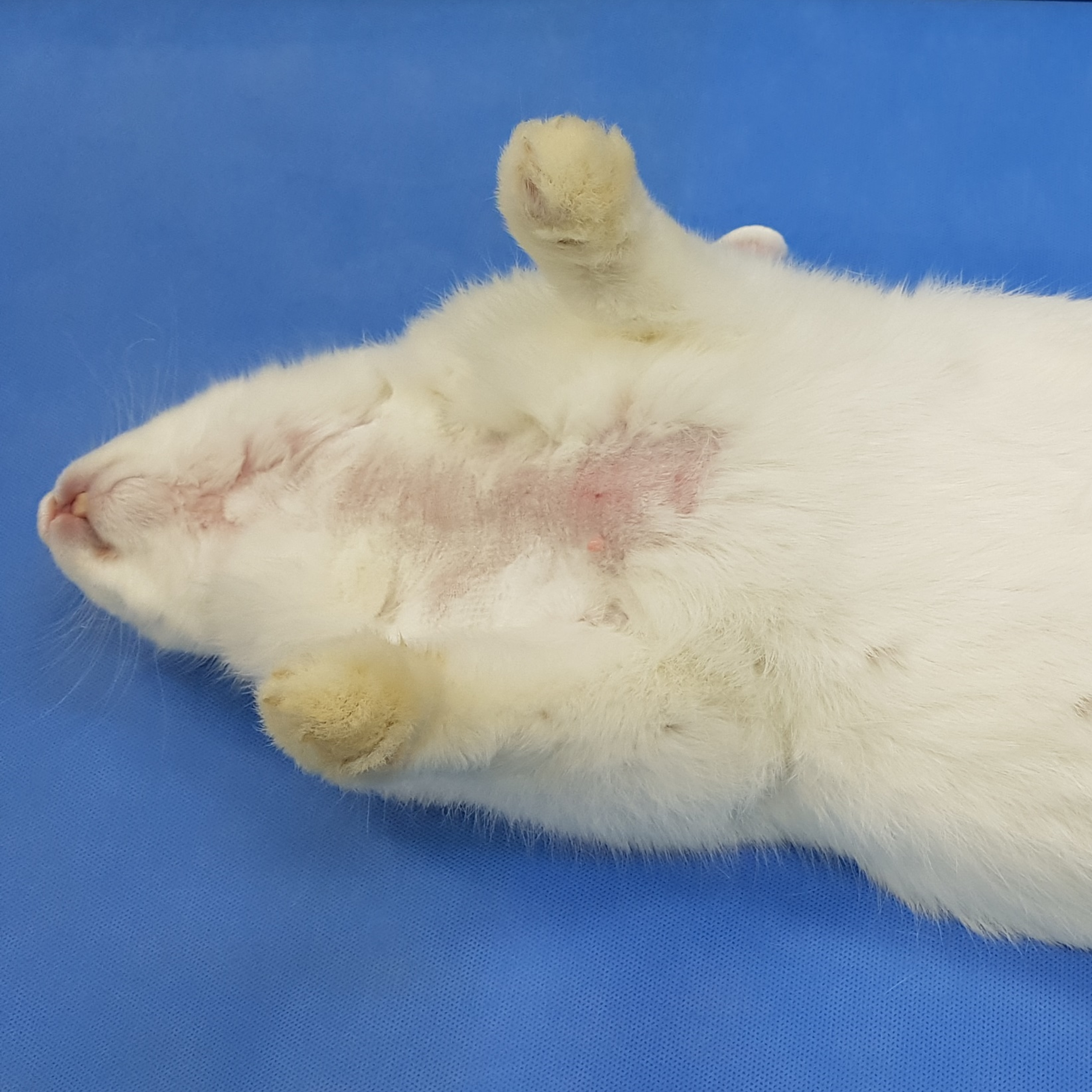

Supplement: Supplementary file 5 — Supplementary Information 1. [file 41598_2022_21896_MOESM5_ESM.tif]
