# Supplementary material for: A rabbit model for embolic infarct potentials of injectables using ultrasound-guided carotid artery puncture
Source: Sci Rep. 2022 Nov 10;12:19269. doi: 10.1038/s41598-022-21896-9 (PMC9649683; doi:10.1038/s41598-022-21896-9)
Supplement: Supplementary file 6 — Supplementary Information 2. [file 41598_2022_21896_MOESM6_ESM.docx]

**A Rabbit Model for Embolic Infarct Potentials of Injectables**

**Using Ultrasound-Guided Carotid Artery Puncture**

Jiwoon Seo MD., Joon Woo Lee MD. PhD., Jungheum Cho MD.,

Eugene Lee MD. PhD., Heung Sik Kang MD. PhD.

**SUPPLEMENTARY INFORMATION**

**Supplementary Figure S1. Rabbit prepared for ultrasound-guided carotid artery puncture.**

**Supplementary Video S2. Ultrasound-guided carotid artery puncture and administration of the embolic agent.**

**Supplementary Video S3. A rabbit showing seizure-like movement after embolic agent administration.**

**Supplementary Video S4. A rabbit showing hemiparetic movement, 24 hours after embolic agent administration.**

**Supplementary Video S5. An abrupt hematoma formation after puncture failure.**
